# Supplementary material for: G1 Cell Cycle Arrest and Extrinsic Apoptotic Mechanisms Underlying the Anti-Leukemic Activity of CDK7 Inhibitor BS-181
Source: Cancers (Basel). 2020 Dec 19;12(12):3845. doi: 10.3390/cancers12123845 (PMC7766600; doi:10.3390/cancers12123845)
Supplement: Supplementary file 1 [file cancers-12-03845-s001.zip › cancers-1018533-Suppl-proof-final.pdf]

Supplementary Materials:

G_1 Cell Cycle Arrest and Extrinsic Apoptotic Mechanisms Underlying the Anti-Leukemic Activity of CDK7 Inhibitor BS-181

Shin Young Park [1], Ki Yun Kim [1], Do Youn Jun [2], Su-Kyeong Hwang [2,3] and Young Ho Kim [1,*]

Supplementary 1.

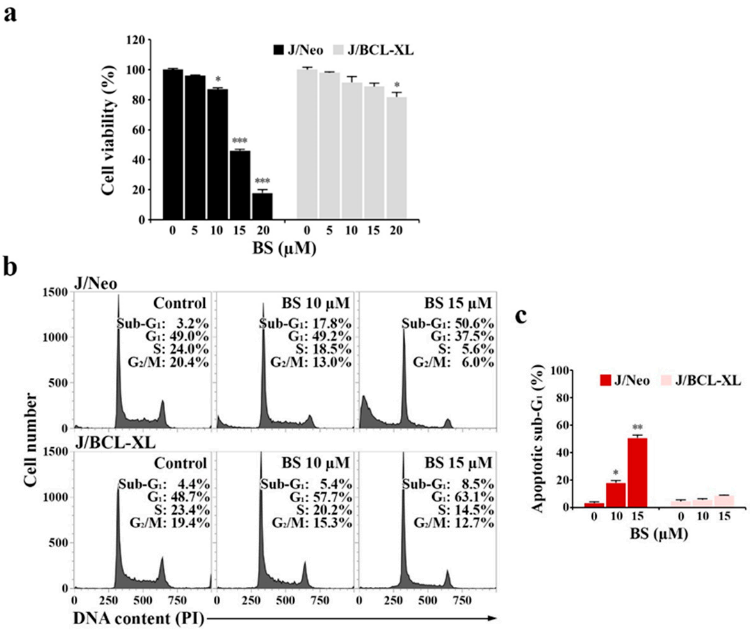

Supplementary Figure S1. Effect of BS-181 on cell viability, cell cycle distribution in Jurkat T cell clones transfected with the empty vector (J/Neo cells) or the *BCL-XL* expression vector (J/BCL-XL cells). (a) Cell viability was determined by incubating each cell type (5×10^4 cells/well) with the indicated concentrations of BS-181 in a 96-well plate for 20 h and with MTT solution for an additional 4 h. Mean ± SD (n = 3 with three replicates per independent experiment). **P* < 0.05, ****P* < 0.005, compared with the control; (b–c) Equivalent cultures were prepared for analysis of cell cycle distribution by flow cytometry as described in Materials and Methods. BS: BS-181. Mean ± SD of triplicate experiments. **P* < 0.05, ***P* < 0.01, compared with the control.

Supplementary 2.

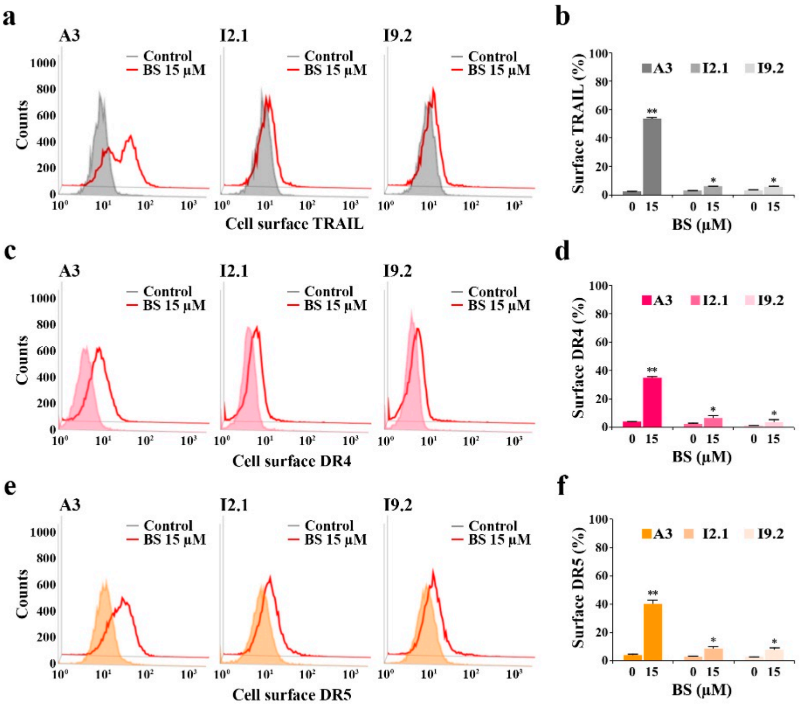

Supplementary Figure S2. Differential effect of BS-181 on cell surface TRAIL, DR4, and DR5 levels in wild-type A3, FADD-deficient I2.1, and caspase-8-deficient I9.2 Jurkat T cell clones. (a-f) After individual Jurkat T cell clones were exposed to 10 or 15 μM BS-181 for 24 h, cells were treated with anti-TRAIL, BV421-conjugated anti-DR4, or Alexa Fluor 647-labeled anti-DR5 antibody for 45 min on ice. The cells treated with anti-TRAIL antibody were rinsed and treated with Alexa Fluor 488-labeled anti-rabbit IgG antibody for an additional 30 min on ice. After fixation in 1% paraformaldehyde/PBS, TRAIL-, DR4-, or DR5-positive cells were measured by flow cytometry. BS: BS-181. A representative result is shown; two additional experiments yielded similar results. Mean ± SD of triplicate experiments. **P* < 0.05, ***P* < 0.01, compared with the control.

Supplementary 3.

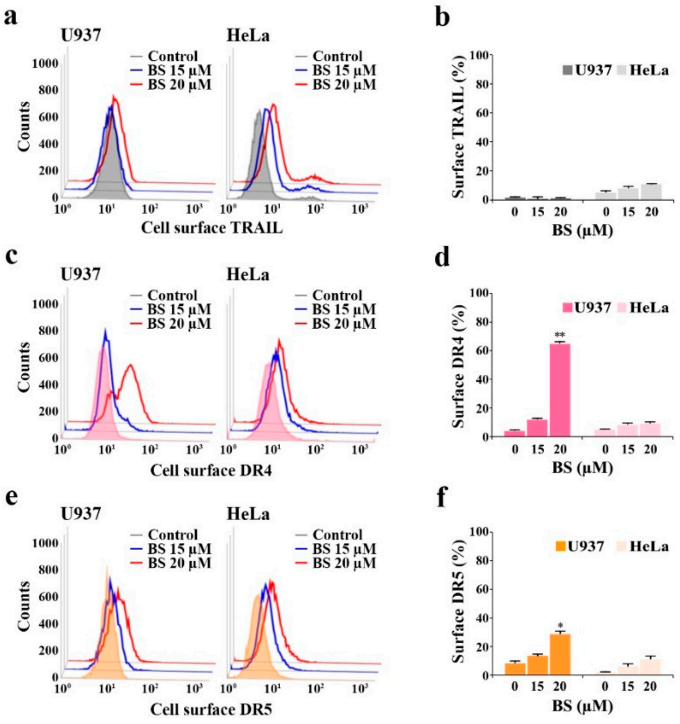

Supplementary Figure S3. Effect of BS-181 on cell surface TRAIL, DR4, and DR5 levels in U937 and HeLa cells. (a-f) After U937 (5×10^5 cells/mL) or HeLa cells (1.7×10^5 cells/mL) were exposed to 15 or 20 μM BS-181 for 24 h, the cells were treat with the anti-TRAIL, BV421-conjugated anti-DR4, or Alexa Fluor 647-labeled anti-DR5 antibody for 45 min on ice. The cells treated with anti-TRAIL antibody were rinsed and treated with Alexa Fluor 488-labeled anti-rabbit IgG antibody for an additional 30 min on ice. After fixation in 1% paraformaldehyde/PBS, TRAIL-, DR4-, or DR5-positive cells were measured by flow cytometry. BS: BS-181. A representative result is shown; two additional experiments yielded similar results. Mean ± SD of triplicate experiments. *$P < 0.05$, **$P < 0.01$, compared with the control.
