# Supplementary material for: G1 Cell Cycle Arrest and Extrinsic Apoptotic Mechanisms Underlying the Anti-Leukemic Activity of CDK7 Inhibitor BS-181
Source: Cancers (Basel). 2020 Dec 19;12(12):3845. doi: 10.3390/cancers12123845 (PMC7766600; doi:10.3390/cancers12123845)

Original western blot data in Fig. 2

e

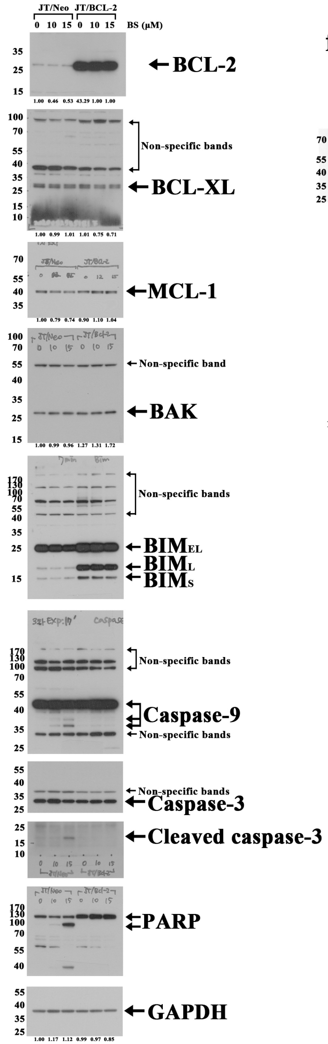

f

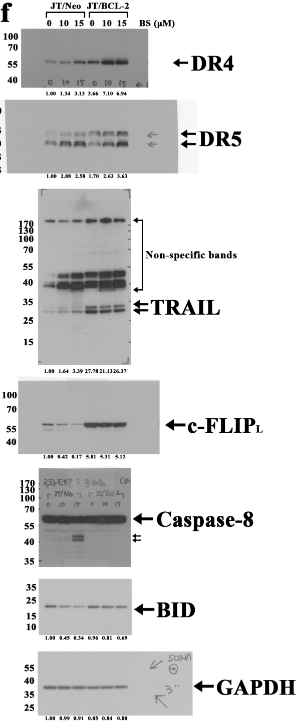

Original western blot data in Fig. 4

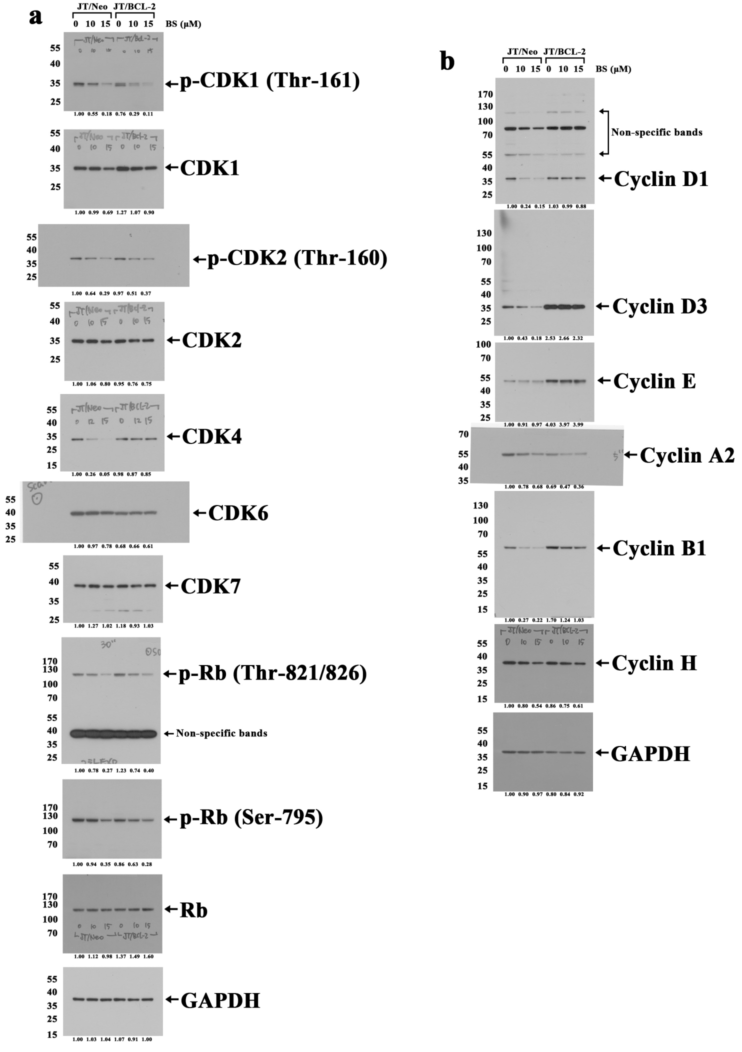

Original western blot data in Fig. 5

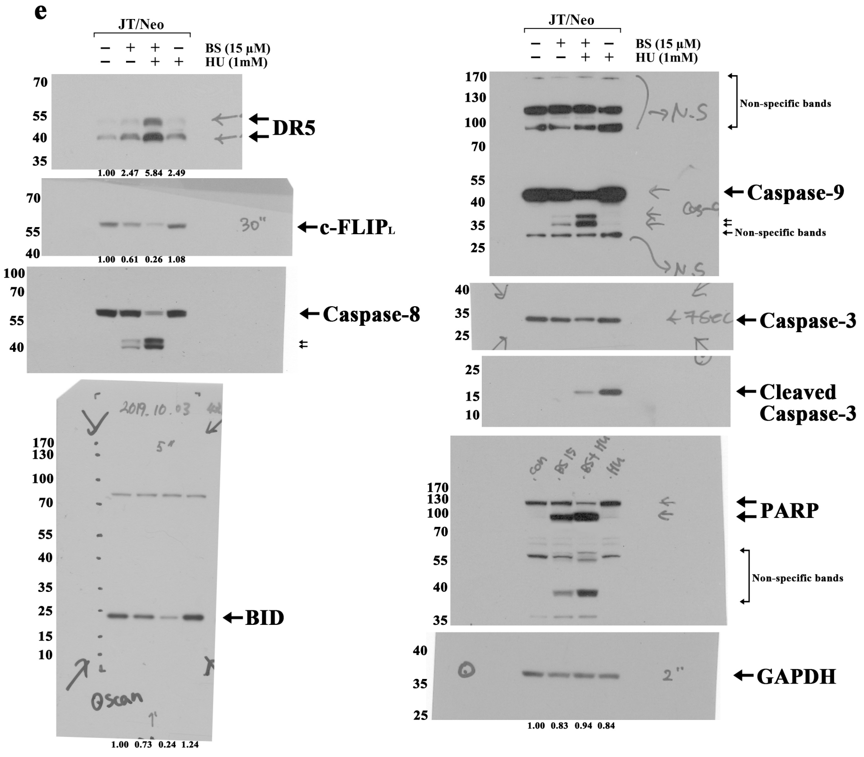

Original western blot data in Fig. 7

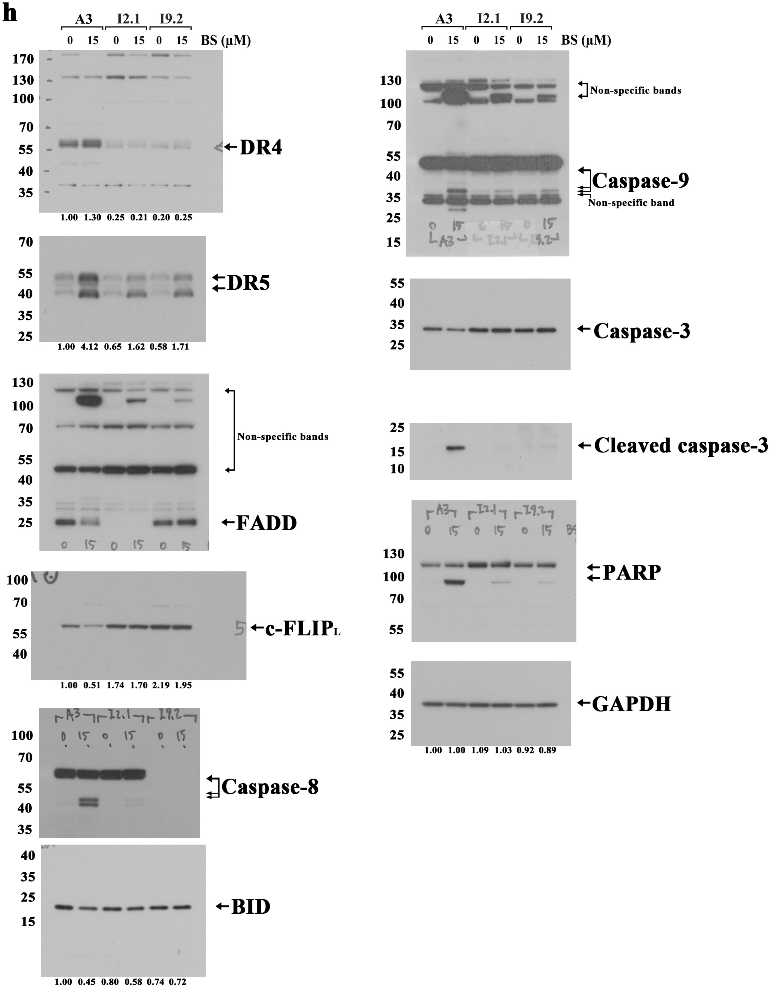

Original western blot data in Fig. 8

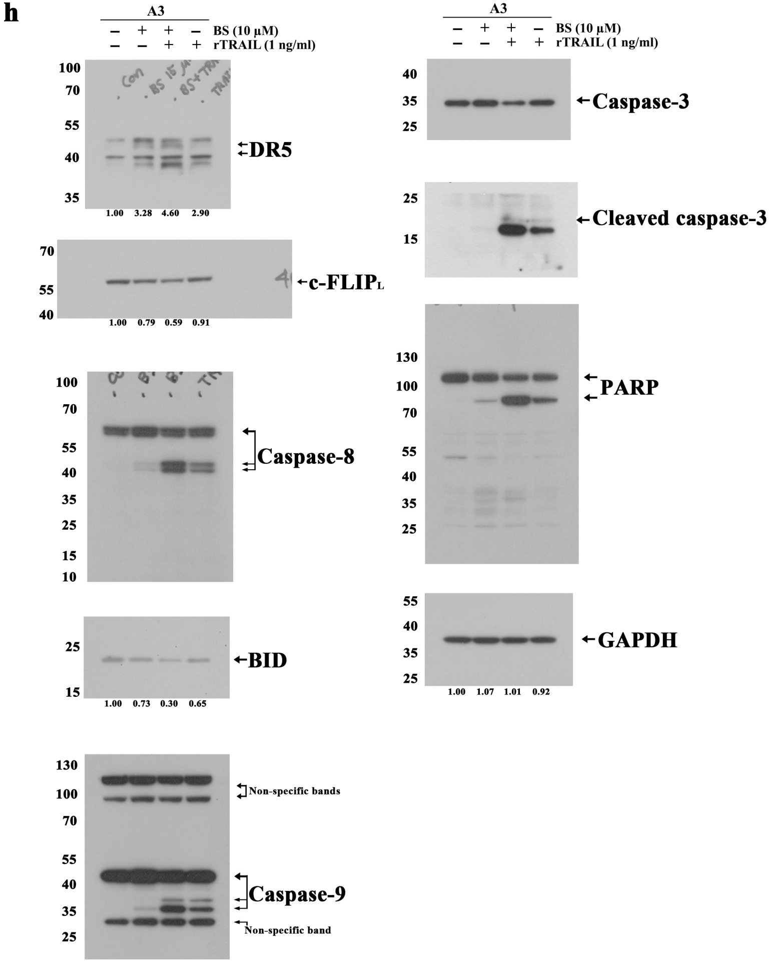

Supplement: Supplementary file 1 [file cancers-12-03845-s001.zip › Original Western blot figures (revised).pdf]
